# Gene analogue finder: a GRID solution for finding functionally analogous gene products

**DOI:** 10.1186/1471-2105-8-329

**Published:** 2007-09-03

**Authors:** Angelica Tulipano, Giacinto Donvito, Flavio Licciulli, Giorgio Maggi, Andreas Gisel

**Affiliations:** 1 Dipartimento Interateneo di Fisica, Università e Politecnico di Bari, via Amendola 173, 70126 Bari Italy; 2 INFN Bari, Via Amendola 173, Bari, Italy; 3 Istituto di Tecnologie Biomediche, CNR, Via Amendola 122/D, Bari, Italy

## Abstract

**Background:**

To date more than 2,1 million gene products from more than 100000 different species have been described specifying their function, the processes they are involved in and their cellular localization using a very well defined and structured vocabulary, the gene ontology (GO). Such vast, well defined knowledge opens the possibility of compare gene products at the level of functionality, finding gene products which have a similar function or are involved in similar biological processes without relying on the conventional sequence similarity approach. Comparisons within such a large space of knowledge are highly data and computing intensive. For this reason this project was based upon the use of the computational GRID, a technology offering large computing and storage resources.

**Results:**

We have developed a tool, G**EN**e Analo**G**ue F**IN**d**E**r (ENGINE) that parallelizes the search process and distributes the calculation and data over the computational GRID, splitting the process into many sub-processes and joining the calculation and the data on the same machine and therefore completing the whole search in about 3 days instead of occupying one single machine for more than 5 CPU years. The results of the functional comparison contain potential functional analogues for more than 79000 gene products from the most important species. 46% of the analyzed gene products are well enough described for such an analysis to individuate functional analogues, such as well-known members of the same gene family, or gene products with similar functions which would never have been associated by standard methods.

**Conclusion:**

ENGINE has produced a list of potential functionally analogous relations between gene products within and between species using, in place of the sequence, the gene description of the GO, thus demonstrating the potential of the GO. However, the current limiting factor is the quality of the associations of many gene products from non-model organisms that often have electronic associations, since experimental information is missing. With future improvements of the GO, this limit will be reduced. ENGINE will manifest its power when it is applied to the whole GODB of more than 2,1 million gene products from more than 100000 organisms. The data produced by this search is planed to be available as a supplement to the GO database as soon as we are able to provide regular updates.

## Background

Gene ontology (GO) [1-4], with the corresponding associations with the gene products, is becoming a very valuable and important form of knowledge in bioinformatics. GO is already frequently used to analyse and cluster results of many bioinformatics applications and especially high-throughput applications [5]. Such tools are mainly used to elaborate an overrepresentation of a few functions and processes in a large list of gene products in order to provide clues as to what the gene products in the list might have in common and to better understand the biological background of the biological experiments under investigation.

The GO model is a directed acyclic graph (DAG) in which the terms and the term-to-term relationships provide the conceptualisation of the biological domain of knowledge [2]. The knowledge is split into three topics (*term types*), namely 'biological process', 'molecular function' and 'cellular component' and therefore into three independent DAGs, used to describe gene products for all organisms. A repository of the current GO terms and gene products with GO associations is frequently compiled in a MySQL database, the GO database (GODB) [3].

Collaborating institutions maintaining the major biological databases provide sets of data consisting of links between the database entries and GO terms, the so-called *associations*. These associations are well documented and characterized by the *type of evidence *supporting the association. High-quality associations are available, structured into 13 classes, normally based on a curatorial review of the literature, experimental evidence or computational analysis methods. Many associations were made using a number of different automatic comparative methods covering both model and less experimentally traceable organisms. More information about evidence classes can be found on the Gene Ontology web site [6].

GO is a recent effort to provide a unique description of gene products from a wide range of different organisms and it is therefore still heterogenic in terms of precision and reliability. Since experimental information is often missing, gene products from less well-studied organisms often inherit low level GO terms from orthologous gene products of well-studied organisms such as human or mouse. Therefore the precision and reliability of such descriptions are much lower and this influences the quality of knowledge of the GO.

However, with the increasing number of gene products described using GO terminology and the daily increase in the precision of their description, GO will play an ever more important role in comparing as well as establishing links between gene products, of the same or across different organisms, on the level of functionality. Finding gene products from different species with similar functionality, or those involved within biological processes similar to the gene product in which they are interested, will benefit researchers in many fields.

GRID computing is a quickly evolving technology driven by several initiatives all over the world. In Europe the EGEE (Enabling Grids for E-sciencE) project [7] is the major driving force, beside smaller but no less important national projects, to build on recent advances in GRID technology and build a service GRID infrastructure which is available to scientists 24 hours a day. In recent years the GRID service has become a technique for users such as physicists, chemists and biologists. In brief, the computational GRID connects under unique middleware a certain number of computers representing the resources for computation and storage. The connection is loose, as all the computers involved are still independent. Jobs are submitted to the GRID by means of the User Interface (UI). After submission, computers dedicated to job submission management, known as resource brokers (RB), distribute the jobs to free computers, the worker nodes (WN), for execution. In this way a large number of computational resources can be formed, such as the EGEE infrastructure connecting more than 25000 CPUs [8]; it is theoretically possible to use all these computational resources for a single problem with a large number of jobs. However, the EGEE infrastructure is divided into virtual organizations (VO), and for biological and biomedical applications a VO 'biomed' has been created, recently reaching a total of more than 5000 CPUs.

In our study we presented G**en**e Analo**g**ue F**in**d**e**r (ENGINE), an application of the GO to compare gene products at the level of their description and to suggest gene products that are functionally analogous to each other. Conventional methods of comparing different gene products are principally based on a comparison at the level of the corresponding sequences. According to the sequence similarity, the gene products are considered similar also in respect to their function. However, the "sequence – function" correlation is only partially applicable. With ENGINE, using the GO, we are able to compile comparisons between gene products on the level of their descriptions, profiting from the large and continuously increasing knowledge about all the annotated gene products.

Such a comparison [SGC1] within such a large amount of gene products already associated to GO terms, and the resulting high number of associations, the search has become a data- and process-demanding task and would occupy our cluster for months or even years. The approach we were followed to solve this resource problem was to distribute the MySQL database GODB over the available computational GRID resources, paralleling the search process and reducing the load on the single computers involved to several hours, processing the total search in about 3 days.

Hence, we have demonstrated the potential of the GO to find functionally analogous gene products and the power of the computational GRID to solve such large data comparisons.

## Results

### The algorithm

#### Path information

To calculate similarity between gene products based on gene ontology (GO), we cannot consider only the associated gene ontology terms (GO terms). Apparently different associated terms might share parental terms with similar global functionality. To profit from this information we need to search for all the parental terms of the associated terms; in other words, we need to build the paths from the associated terms to the root of the tree, and then compare the whole paths of associated GO terms between two gene products. All the path's GO terms different from the directly associated ones are called *indirectly associated terms*. The advantage of this approach over a previously proposed one in which only the first common parent was used for the comparison [9] lies in the observation that a longer path characterizing an associated GO term corresponds to better knowledge of the specific gene product as well as the corresponding functions and processes the gene product is involved in. Using all members in the path for a comparison of two gene products, the similarity value increases when more terms are shared between the two gene products, but it also decreases when many terms not in common are present. With this approach of using the path for the comparison we take into account all the known knowledge. However, just comparing the number of common and non-common terms is not an accurate approach, since the information content of different GO terms is different and should be weighted accordingly.

#### Semantic similarity measurement

Moving along the path of a specific GO term away from the root, the detail of the information of a given term increases from node to node. The specificity of information is directly dependent upon the level of the node within the path, but it is not comparable between two different paths. A GO term at level 6 in one path can be much less descriptive than a term at the same level in another path. Therefore, using the level within a path as a measurement of similarity is a poor technique. One method of determining the semantic similarity of two terms is to examine how frequently those terms are used to describe different gene products [9,10] considering the notion of 'information content'. The less a term of a vocabulary is used the higher the information content and the more descriptive it is.

We therefore calculated the probability p(term) for every term by counting the number of gene products associated with a term or any of its children, divided by the number of total associations between the GO terms and gene products. Information content (detail level of the description) is inversely proportional to p(term) (Table 1). The highest p(term) values are found for GO terms 'molecular function', 'biological process' and 'cellular component', which are the roots of the three DAGs, however, their p(term) value is not 1 (Table 1), since not every gene product has an association with all three *term types*. Table 1 demonstrates that terms of the same level in different paths can have clearly different information content and therefore different p(term) values. The p(term) value was then used as a weight within the statistical analysis and entered as 1-p(term) to obtain an additive weight system for high 'information content' GO terms.

**Table 1:** Examples of p(term) values

name	level	term-type	count	p(term)
molecular_function	1	molecular_function	3471081	0.477303
biological_process	1	biological_process	2243629	0.308518
cellular_component	1	cellular_component	1864423	0.256374
hormone activity	4	molecular_function	4504	0.000619338
gliogenesis	4	biological_process	95	1.30633e-05
cell fate specification	5	biological_process	204	2.80517e-05
angiogenesis	7	biological_process	363	4.99156e-05

Examples of GO terms and their level in the tree are listed including their frequency of use (count) to describe gene products and the corresponding p(term) value.

#### Statistics for comparison

The non-parametric χ^2 ^– test is a typical statistical test used to compare two different samples, in our case two gene products A and B, according to some characteristics or aspects, in our case the GO terms directly or indirectly associated with the gene products. Since every gene product can be described using a different number of GO terms, the table of values was designed as a 2 × 2 array containing the sum of 1-p(term) of present or absent GO terms according to the two compared gene products. (Table 2) [11]. In this way we have the same degree of freedom for all gene-product-to-gene-product comparisons; therefore the χ^2 ^– values are comparable. All three term-types were taken into account for the main search in order to increase the total description and to enable separation of, for example, two gene products with the same function acting in different cellular components. However, we also calculated the statistics for each separate *term-type *to have a 'molecular function', 'biological process' and 'cellular component' specific comparison to find functional analogues based only on one term-type. This is an example of how our method can be used to find gene products exhibiting a particular functionality which is independent of their cellular localization.

**Table 2:** The 2 × 2 array for the χ^2 ^calculation

	Terms present in gene product A	Terms not present in gene product A
Terms present in gene product B	O11=(∑all termscommonto A and B(1−p(term))) MathType@MTEF@5@5@+=feaafiart1ev1aaatCvAUfKttLearuWrP9MDH5MBPbIqV92AaeXatLxBI9gBaebbnrfifHhDYfgasaacH8akY=wiFfYdH8Gipec8Eeeu0xXdbba9frFj0=OqFfea0dXdd9vqai=hGuQ8kuc9pgc9s8qqaq=dirpe0xb9q8qiLsFr0=vr0=vr0dc8meaabaqaciaacaGaaeqabaqabeGadaaakeaacqWGpbWtdaWgaaWcbaGaeGymaeJaeGymaedabeaakiabg2da9maabmaabaWaaabuaeaacqGGOaakcqaIXaqmcqGHsislcqWGWbaCcqGGOaakcqWG0baDcqWGLbqzcqWGYbGCcqWGTbqBcqGGPaqkcqGGPaqkaSabaeqabaGaemyyaeMaemiBaWMaemiBaWMaeeiiaaIaemiDaqNaemyzauMaemOCaiNaemyBa0Maem4CamhabaGaem4yamMaem4Ba8MaemyBa0MaemyBa0Maem4Ba8MaemOBa4gabaGaemiDaqNaem4Ba8MaeeiiaaIaemyqaeKaeeiiaaIaemyyaeMaemOBa4MaemizaqMaeeiiaaIaemOqaieaaeqaniabggHiLdaakiaawIcacaGLPaaaaaa@6050@	O12=(∑all termspresent in Bbut not in A(1−p(term))) MathType@MTEF@5@5@+=feaafiart1ev1aaatCvAUfKttLearuWrP9MDH5MBPbIqV92AaeXatLxBI9gBaebbnrfifHhDYfgasaacH8akY=wiFfYdH8Gipec8Eeeu0xXdbba9frFj0=OqFfea0dXdd9vqai=hGuQ8kuc9pgc9s8qqaq=dirpe0xb9q8qiLsFr0=vr0=vr0dc8meaabaqaciaacaGaaeqabaqabeGadaaakeaacqWGpbWtdaWgaaWcbaGaeGymaeJaeGOmaidabeaakiabg2da9maabmaabaWaaabuaeaacqGGOaakcqaIXaqmcqGHsislcqWGWbaCcqGGOaakcqWG0baDcqWGLbqzcqWGYbGCcqWGTbqBcqGGPaqkcqGGPaqkaSabaeqabaGaemyyaeMaemiBaWMaemiBaWMaeeiiaaIaemiDaqNaemyzauMaemOCaiNaemyBa0Maem4CamhabaGaemiCaaNaemOCaiNaemyzauMaem4CamNaemyzauMaemOBa4MaemiDaqNaeeiiaaIaemyAaKMaemOBa4MaeeiiaaIaemOqaieabaGaemOyaiMaemyDauNaemiDaqNaeeiiaaIaemOBa4Maem4Ba8MaemiDaqNaeeiiaaIaemyAaKMaemOBa4MaeeiiaaIaemyqaeeaaeqaniabggHiLdaakiaawIcacaGLPaaaaaa@6A6E@
Terms not present in gene product B	O21=(∑all termspresent in Abut not in B(1−p(term))) MathType@MTEF@5@5@+=feaafiart1ev1aaatCvAUfKttLearuWrP9MDH5MBPbIqV92AaeXatLxBI9gBaebbnrfifHhDYfgasaacH8akY=wiFfYdH8Gipec8Eeeu0xXdbba9frFj0=OqFfea0dXdd9vqai=hGuQ8kuc9pgc9s8qqaq=dirpe0xb9q8qiLsFr0=vr0=vr0dc8meaabaqaciaacaGaaeqabaqabeGadaaakeaacqWGpbWtdaWgaaWcbaGaeGOmaiJaeGymaedabeaakiabg2da9maabmaabaWaaabuaeaacqGGOaakcqaIXaqmcqGHsislcqWGWbaCcqGGOaakcqWG0baDcqWGLbqzcqWGYbGCcqWGTbqBcqGGPaqkcqGGPaqkaSabaeqabaGaemyyaeMaemiBaWMaemiBaWMaeeiiaaIaemiDaqNaemyzauMaemOCaiNaemyBa0Maem4CamhabaGaemiCaaNaemOCaiNaemyzauMaem4CamNaemyzauMaemOBa4MaemiDaqNaeeiiaaIaemyAaKMaemOBa4MaeeiiaaIaemyqaeeabaGaemOyaiMaemyDauNaemiDaqNaeeiiaaIaemOBa4Maem4Ba8MaemiDaqNaeeiiaaIaemyAaKMaemOBa4MaeeiiaaIaemOqaieaaeqaniabggHiLdaakiaawIcacaGLPaaaaaa@6A6E@	O22=(∑all terms(1−p(term))−∑all termsin A or B(1−p(term))) MathType@MTEF@5@5@+=feaafiart1ev1aaatCvAUfKttLearuWrP9MDH5MBPbIqV92AaeXatLxBI9gBaebbnrfifHhDYfgasaacH8akY=wiFfYdH8Gipec8Eeeu0xXdbba9frFj0=OqFfea0dXdd9vqai=hGuQ8kuc9pgc9s8qqaq=dirpe0xb9q8qiLsFr0=vr0=vr0dc8meaabaqaciaacaGaaeqabaqabeGadaaakeaacqWGpbWtdaWgaaWcbaGaeGOmaiJaeGOmaidabeaakiabg2da9maabmaabaWaaabuaeaacqGGOaakcqaIXaqmcqGHsislcqWGWbaCcqGGOaakcqWG0baDcqWGLbqzcqWGYbGCcqWGTbqBcqGGPaqkcqGGPaqkaSqaaiabdggaHjabdYgaSjabdYgaSjabbccaGiabdsha0jabdwgaLjabdkhaYjabd2gaTjabdohaZbqab0GaeyyeIuoakiabgkHiTmaaqafabaGaeiikaGIaeGymaeJaeyOeI0IaemiCaaNaeiikaGIaemiDaqNaemyzauMaemOCaiNaemyBa0MaeiykaKIaeiykaKcalqaabeqaaiabdggaHjabdYgaSjabdYgaSjabbccaGiabdsha0jabdwgaLjabdkhaYjabd2gaTjabdohaZbqaaiabdMgaPjabd6gaUjabbccaGiabdgeabjabbccaGiabd+gaVjabdkhaYjabbccaGiabdkeacbaabeqdcqGHris5aaGccaGLOaGaayzkaaaaaa@71F5@

The 2 × 2 array used for the χ^2 ^– calculation to compare gene product A with gene product B.

To reduce the vast amount of output data, we stored only the best 100 hits in the output file, including, according to a pre-evaluation of the algorithm (data not shown), the most significant candidates for a functional analogy. The best 100 hits were chosen by sorting the components of the total output list by their χ^2 ^– squared values and selecting the 100 highest values.

### The computing

#### Data set

The version of the GO database (GODB) used for the present study (version 2005-11-1) contained 20088 terms within the three trees. Those terms described more than 1760000 gene products with more than 7200000 associations.

To ensure that for all gene products we found the best functional analogues within the whole GODB, the GO terms of one gene product should have been compared with all other gene products in the GODB. However, many gene products are associated with only a few and/or low 'information content' GO terms. A previous test run comparing more than 100000 gene products showed that those with fewer than 15 directly or indirectly (parents) associated GO terms should not be considered by the search for the first 100 best hits (unpublished data). For this reason, all gene products with 15 or fewer directly or indirectly associated GO terms were eliminated from both the input list and the total list of gene products with GO terms associated. With this restriction, the total gene product list of 1763776 entries was drastically reduced to 1076279 gene products (61% of total). Our input list included all gene products related to 13 fully sequenced organisms annotated within Ensembl [12], namely *Caenorhabditis elegans, Caenorhabditis briggsae, Anopheles gambiae *(African malaria mosquito), *Drosophila melanogaster *(fruit fly), *Apis mellifera *(honey bee), *Danio rerio *(zebrafish), *Gallus gallus *(chicken), *Pan troglodytes *(chimpanzee), *Homo sapiens *(human), *Mus musculus *(house mouse), *Rattus norvegicus *(Norway rat), *Takifugu rubripes*, and *Tetraodon biocellatus*. A total of 125791 gene products in the GODB belong to these organisms. Applying the '15 directly or indirectly associated GO terms' rule results in a final input list of 79125 gene products (63% of candidates). Therefore the search compares 79125 with 1076279 gene products, resulting in more than 85 billion comparisons.

#### Computational solution

The algorithm for the statistical comparison of two gene products according to their description was implemented in Perl. The necessary information was retrieved by accessing the GODB by means of the Perl 'DBI' modules, while the 'Statistics_chisquare' module was used for the χ^2 ^– calculation [13]. The analysis of one gene product against all other selected gene products occupies one CPU (1.0 GHz Pentium III) for between 30 and 45 min., depending on the number of associated GO terms. The analysis of all 79125 gene products would require approximately 5 CPU-years.

Due to the enormous quantity of computer resources available, the GRID enables splitting a large, complex application into many smaller jobs running in parallel, greatly reducing execution time. For this specific case, however, splitting the full search into several smaller jobs does not increase the speed of the search, because the limiting factor is the limited bandwidth of the single database which contains all the required information. Improving the performance of the search application will require increasing the number of data sources; the best possible performance will be obtained by providing a database server for each job running on the GRID.

An important new capacity of ENGINE is its ability to distribute and temporally install a relational database such as MySQL on the same WN on the GRID where ENGINE is running. In most applications simple files are distributed and used for calculation. ENGINE can distribute and install on each WN the GODB downloaded from the godatabase site without further modification, thus reducing maintenance time for the application. A locally installed database not only drastically reduces processing time, since there is no competition between several parallel queries, but also there is and no network transition time, which is very important for data-access intensive processes.

The INFN production GRID [14] was used for the search. Its primary scope is to provide computational resources to INFN high-energy physics experiments, but it is also open to other sciences. It is part of the EGEE infrastructure, comprising more than 20 sites all over Italy and, at the time of the search, integrating more than 1500 CPUs, mostly Pentium IIIs and IVs or equivalents, all running the Linux Red Hat 7.3 operating system. Most of the farms were using Open-PBS [15] as their local batch system, while some used LSF [16]. The GRID middleware was LCG2.6 [17].

That functionally equivalent computers, all running the same operation system, were used throughout the GRID infrastructure helped simplify the database installation procedure; a static linked binary MySQL server was used. To guarantee faster data installation, the database content was transferred from the central repository to the worker nodes using the MySQL binary format. Before the actual search job was started, a bash script, launched on the WN, performed the entire installation procedure by executing all needed operations in a well-defined order and by making all necessary checks to verify whether the created environment was adequate to run the job.

After installing the database, ENGINE installs and sets up the Perl libraries for use by the ENGINE Perl script. Since the use of Perl and its modules is very common in bioinformatics, this type of distributed installation may also be of interest for other applications.

The installation procedure described above was executed without incident on all sites of the INFN production GRID infrastructure which support virtual organization (VO) "bio".

#### Submitting the job to the GRID

To perform the analogous genes search, the entire list of reference gene products (79125) was split into sub-lists of 20 gene products each. The number of gene products per job was chosen to produce jobs with execution times short enough to avoid problems due to accidental hardware or software crashes, but long enough to keep environment set-up time negligible compared to the total job execution time. With the chosen number of genes, the jobs lasted ten hours on average, while environment set up time was on the order of only 6% of job execution time.

The jobs were submitted by means of a Perl script, running on the User Interface (UI), which first created the job input file with the sub-list of gene products assigned to that particular job by splitting the full list of reference gene products. Then the job description file (jdl file) was created with appropriate input and output file names, which varied from job to job for bookkeeping reasons. Finally, the script consecutively submitted each job, with its corresponding input file, bash script, and jdl file, for parallel execution. It also initialized the monitoring of the job by activating JAM, a Job and Application Monitoring tool [18]. JAM made it possible to follow the steps performed by the bash script during the WN environment installation phase and to keep track in real time of all gene products already examined by all jobs running on the GRID. JAM registered all failed comparisons, which were then re-submitted to successfully complete the full search.

At fixed time intervals, output files produced by completed jobs were retrieved and checked for missing information caused by job failures. A different Perl script was used to identify the missing genes, to generate new lists with those gene products and to re-submit jobs to complete the whole search.

**Figure 1:**
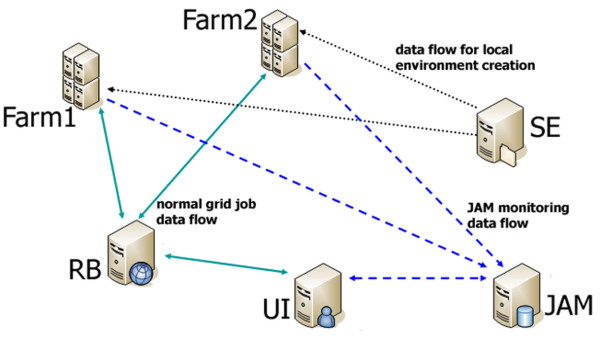
**Job submission schema and data flow**. A job is initiated at the user interface (UI) and forwarded by the resource broker (RB) to the worker nodes or farms. In parallel a job monitoring procedure (JAM) is started to test the success of the executed job. The execution on the farms firstly initiates a download of the required data from a storage element (SE) followed by the effective computing. Then the results can be recovered from the farm via the UI.

In Figure 1 a schematic view of the different processes is shown, including job submission, creation of the local running environment (MySQL server installation and database population), job monitoring (by JAM) and data retrieval.

The search for the analogous candidates for the list of reference gene products (79125) required the submission of a total of 5200 jobs to the GRID, each with 20 gene products as search input. The jobs were distributed to as many as 950 WN assigned by the Resource Broker. Failed jobs (about 16%) were restarted. Total computing time was more than 52000 CPU hours (~ 6 CPU years), including the time needed to set up the running environment, data base and library installations (6% of overall job length). The search was completed in about three days. The acceleration of the process was about 580 times, taking into account the re-running of failed processes, the time for necessary installations per job, and that not all those 950 WN were available at any time.

### Significance of the search results

To demonstrate the value of the search results, we performed a variety of different analyses on the data. One measure of how well a gene product is described, among others, is the number of other gene products having exactly the same description; the description of a gene product is the non-redundant list of all directly and indirectly associated GO terms. We divided the results into three groups according to the number of identically annotated gene products. Group 1 (26587; 33.6%) comprised those analyzed gene products which had a unique description. In group 2 (10251; 13.0%), the analyzed gene products shared exactly the same description with fewer than 100 other gene products, and in group 3 (42286; 53.4%), more than 100 other gene products. About half of the gene products (groups 1 and 2) have quite detailed descriptions, which makes it possible to distinguish them from the majority of the analysed gene products. This proportion should increase for each new GODB release, since the list of GO terms is increasing and, more importantly, the quality and the number of associations are improving.

**Figure 2:**
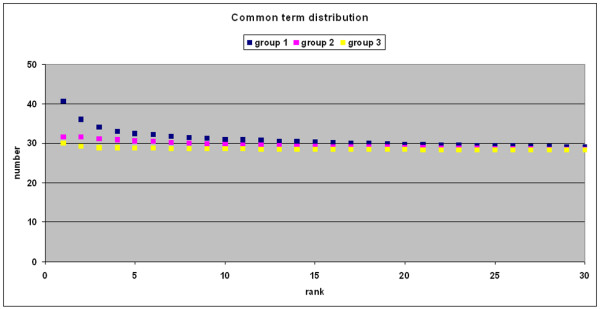
**Common term distribution**. The average number of common terms between the input gene product and the analogous gene product found is plotted against the ranking of hits. Group 1 members are plotted in yellow, group 2 in purple, and group 3 in blue. x-axis: Ranking of the search hits according the χ^2 ^– value; y-axis: Average of total common terms between the input gene product associations and the hit gene product associations.

Another measure of the quality of the description is the total number of directly and indirectly associated terms (parents). As mentioned before, as the total number of directly and indirectly associated terms increases, the gene product's description becomes more detailed; therefore, the more common terms two gene products share, the higher the quality of the comparison. The average number of common terms in group 1 is higher than within the other groups but decreases with increasing ranking number of the hits (Figure 2). However, the first 25 ranks still have a higher average number of common terms than groups 2 and 3, demonstrating that the quality of comparison is higher in group 1 than in groups 2 and 3.

The same analysis, carried out individually for *term types *'molecular function' and 'biological process', clearly showed that the combination of the three *term types *produces a much better description than any *term type *alone. For example, by using only *term type *'biological process', the search found that 95% (75442) of the input gene products were described by GO terms of type 'biological process'. Of this number, only 17.2% (12943) belonged to group 1, 14.0% (10571) to group 2 and the majority, 68.8% (51928) to group 3. By using only *term type *'molecular function', the corresponding values were as follows: 94% (74088) had association terms of type 'molecular function', 12.3% (9092) of which belonged to group 1, 20.1% (14882) to group 2, and 67.6% (50114) to group 3. Limiting the comparison to only a single *term type*, the number of significantly described gene products (groups 1 and 2) dropped drastically. However, we expect that the improvement of the GO and its associations will increase the percentage of well described gene products, i.e., those falling in groups 1 and 2.

These latter results also confirmed the success of the '15 directly or indirectly associated GO terms' rule to eliminate the low level described gene products before the search; most of the gene products have associations with GO terms of all *term types*. Of the total number of gene products covering the chosen species, only 89% had an association to *term type *'molecular function' terms and only 76% to *term type *'biological process' terms. Those values were increased to 94% and 95%, respectively, when the '15 directly or indirectly associated GO terms' rule was applied.

Another measure of the quality of the search results is an analysis of the frequency of use of the involved *types of evidence*. There are 13 different codes for the *type of evidence *validating the GO association, of which 8 are based on experimental information and 3 on computational methods (Table 3). The more *types of evidence *based on experimental information are involved in our comparative analysis, the more the search results differ from computational methods based on sequence comparison, and the more biological information influences our functional analogy search.

**Table 3:** Evidence description and codes

Description	Code	
Inferred by Curator	IC	experimental
Inferred from Direct Assay	IDA	experimental
Inferred from Electronic Annotation	IEA	computational
Inferred from Expression Pattern	IEP	experimental
Inferred from Genetic Interaction	IGI	experimental
Inferred from Mutant Phenotype	IMP	experimental
Inferred from Physical Interaction	IPI	experimental
Inferred from Sequence or Structural Similarity	ISS	computational
Non-traceable Author Statement	NAS	experimental
No biological Data available	ND	--
Inferred from Reviewed Computational Analysis	RCA	computational
Traceable Author Statement	TAS	experimental
Not Recorded	NR	--

Description of the evidence code and assignment to experimental or computational methods.

**Figure 3:**
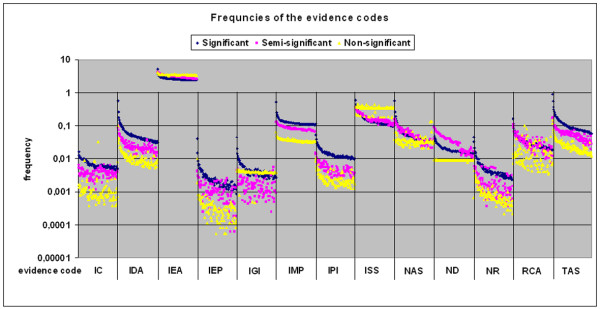
**Frequency of evidence codes**. The average frequencies of use of the 13 evidence codes are plotted against the ranking of the hits. For every evidence code the frequencies were split according to the three groups 1 (blue), 2 (purple) and 3 (yellow). x-axis: evidence code and the corresponding first 100 ranks for each evidence code (see Table 2), y-axis: frequency of usage of the evidence codes.

A graphic was compiled with an average frequency of use of every *type of evidence *code at every position of the ranking of the best 100 hits (Figure 3). This graph demonstrates that the evidence 'Inferred from Electronic Annotation' (code IEA) is the most used *type of evidence *and that it is equally applied throughout all three groups we defined above. The IEA evidence is critical, since it can add valuable additional information to the gene product description, but it can also add incorrect information due to incorrect assumptions. However, it is more important that the frequency of use of all experimental *evidence codes *such as 'Traceable or Non-traceable Author Statements' (TAS or NAS) are significantly higher in the first two groups to validate our results in those groups. Furthermore, the frequency of use drops towards the end of the ranking, indicating a decrease not only in the similarity but also in the specificity and number of descriptions. In group 3, *evidence codes *such as IEA and ISS are more prominent, pointing out that the electronically associated GO terms are overrepresented in gene products with poorer descriptions. In group 2, most of the frequencies of use of the experimental *evidence *behave similarly to the group 1 members and therefore can be evaluated as significantly described gene products. This *evidence code *usage speaks in favour of our analysis of analogous gene products, showing that the significant hits (groups 1 and 2) are dominated by experimental evidence and that they clearly distinguish their information content from sequence comparison.

Both analyses of the nature of the GO show that the state of knowledge is mature for running comparative analysis but that it is not yet sufficient to efficiently include data from the whole set of gene products. However, and more importantly, we demonstrate that the GO has the potential to harbour knowledge for such an analysis.

**Figure 4:**
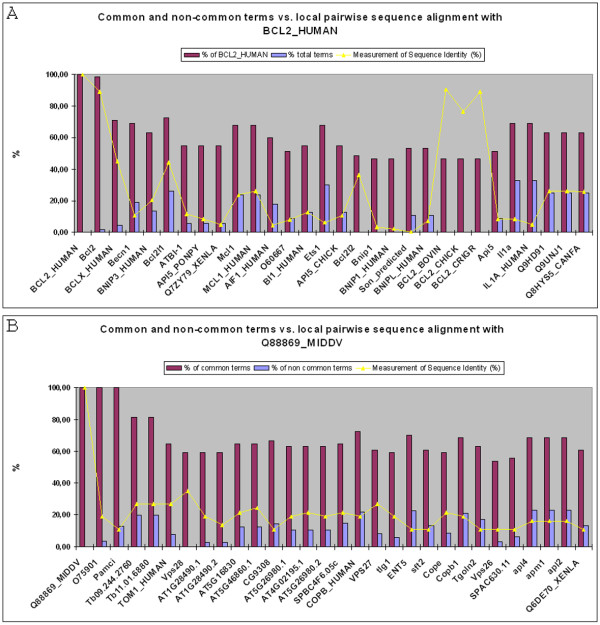
**Sequence identity versus number of common and non-common GO terms**. A comparison of the sequence similarity is plotted together with the percentage of common terms (purple columns) and the non-common terms (blue columns) of potential functional analogues of a) BCL2_HUMAN and b) Q88869_MIDDV. The percentages of the common terms are in relation to the number of terms associated to a) BCL2_HUMAN and b) Q88869_MIDDV, whereas the percentage of the non-common term is calculated in relation to the total associated terms of the same gene product. x-axis: gene products with decreasing ranking; y-axis: percentage of common or non-common terms and percentage of sequence identity.

For quality control on a biological level, we analyzed two gene products: BCL2_HUMAN [UniProt: P10415], a well studied apoptosis gene; and Q88869_MIDDV [UniProt: Q88869] a non-structural polyprotein gene of a mosquito alphavirus, the Middelburg virus. The latter represents a well described gene product of a non well known organism, which adds valuable information to a functional analogue search. We limited this analysis to the 30 best hits. Table 4 lists a collection of information describing the biology of the 30 best suggested analogous gene products of BCL2_HUMAN. Firstly, BCL2_HUMAN was found as the only best hit and therefore is a well-described gene product. Within the 30 best hits we found 12 other gene products which, according to the protein family database (Pfam) [19,20], belong to the same family, the Bcl-2 family. 4 gene products belong to another apoptosis protein family, the apoptosis inhibitory protein 5 (API5) family. This demonstrates how ENGINE can find members of the same or of a similar protein family, which is an important confirmation of our algorithm. It is important to mention that members of gene families within Pfam are assigned by sequence similarity and therefore can be found by sequence based approaches. Focusing on the Bcl-2 family, ENGINE found 5 out of 6 BCL2 orthologous gene products present in the SwissProt database [21-23] with high sequence similarity (Figure 4a, line). Only the mouse orthologue was missing. It seems that there were problems in the mouse association data set of the GODB we used; therefore, it was hard to find any mouse analogues in any search. This problem will be resolved by a future update of our search results, which will use a new version of the GODB.

**Table 4:** Example with BCL2_HUMAN

Ranking	Symbol	UniProt	χ^2^	Common terms	Pfam	Organism	Local alignment	
							hit size	Identity %
1	BCL2_HUMAN	P10415	20080,7	62	Bcl-2	Homo sapiens	239	100.0
2	Bcl2	P49950	19405,8	61	Bcl-2	Rattus norvegicus	239	89.1
3	BCLX_HUMAN	Q07817	13329,9	44	Bcl-2	Homo sapiens	238	45.0
4	Becn1	Q91XJ1	10944,1	43	APG6	Rattus norvegicus	108	23.1
5	BNIP3_HUMAN	Q12983	10529	39	BNIP3	Homo sapiens	263	18.3
6	Bcl2l1	P53563	10426,8	45	Bcl-2	Rattus norvegicus	238	44.5
7	ATBI-1	Q9LD45	10199,5	34	UPF0005	Arabidopsis thaliana	152	18.4
8	API5_PONPY	Q5R644	10015,8	34	API5	Pongo pygmaeus	80	25.0
9	Q7ZY79_XENLA	Q7ZY79	10015,8	34	API5	Xenopus laevis	36	30.6
10	Mcl1	Q9Z1P3	9987,1	42	Bcl-2	Rattus norvegicus	185	30.3
11	MCL1_HUMAN	Q07820	9987,1	42	Bcl-2	Homo sapiens	265	23.4
12	AIF1_HUMAN	P55008	9494,6	37	-	Homo sapiens	33	30.3
13	O60667	O60667	9365,6	32	-	Homo sapiens	50	38.0
14	BI1_HUMAN	P55061	9345,2	34	UPF0005	Homo sapiens	155	19.4
15	Ets1	P41156	9302,5	42	Ets	Rattus norvegicus	53	28.3
16	API5_CHICK	Q5ZMW3	9245,1	34	API5	Gallus gallus	107	23.4
17	Bcl2l2	P70345	9232,3	30	Bcl-2	Mus musculus	235	37.0
18	Bnip1	Q8VHI8	9206,8	29	Sec20	Rattus norvegicus	25	32.0
19	BNIP1_HUMAN	Q12981	9206,8	29	Sec20	Homo sapiens	13	38.5
20	Son_predicted	Q6PDU3	9160,2	33	-	Rattus norvegicus		
21	BNIPL_HUMAN	Q7Z465	9114,8	33	-	Homo sapiens	73	23.3
22	BCL2_BOVIN	O02718	9109,8	29	Bcl-2	Bos taurus	239	90.4
23	BCL2_CHICK	Q00709	9109,8	29	Bcl-2	Gallus gallus	246	74.4
24	BCL2_CRIGR	Q9JJV8	9109,8	29	Bcl-2	Cricetulus griseus	239	89.1
25	Api5	O35841	9083,1	32	API5	Mus musculus	80	25.0
26	Il1a	P16598	9080,0	43	IL1	Rattus norvegicus	92	21.7
27	IL1A_HUMAN	P01583	9077,8	43	IL1	Homo sapiens	42	26.2
28	Q9HD91	Q07820	9060,0	39	Bcl-2	Homo sapiens	265	23.4
29	Q9UNJ1	Q07820	9060,0	39	Bcl-2	Homo sapiens	265	23.4
30	Q8HYS5_CANFA	Q8HYS5	9060,0	39	Bcl-2	Canis familiaris	215	28.4

The table contains the following information: The ranking of the potential functional analogues of BCL2_HUMAN according the χ^2 ^– value, the gene name and the UniProt accession number, the χ^2 ^– value, the number of common terms between a hit and BCL2_HUMAN, the Pfam name with short domain description the gene product belongs to, the organism the gene products is from, and the local alignment characteristics, such as hit length and the % identity of the hit, of the alignment between BCL2_HUMAN and the hit sequence.

Most Bcl2 orthologues, however, have a significantly lower-than-maximum χ^2 ^– value. Figure 4a shows the number of common (purple columns) and non-common (blue columns) terms and indicates that those orthologues do not have nearly as many associated terms as do the BCL2_HUMAN and the rat orthologues. An examination of the evidence code for these associations shows that the orthologous ones inherited the GO terms from well described orthologues, often via common functional domains, and are not yet as well described by experimental evidence as are the human and rat Bcl2. Figure 4a also shows that most of the gene products within the first 30 ranks have 50% or more terms in common with BCL2_HUMAN but the χ^2 ^– value is reduced because of the high number of non-common terms pointing out other functions than those shared with BCL2_HUMAN.

Among the 13 non-Bcl-2 and non-API5 gene products, 8 are, according to their gene descriptions, related to apoptosis, 1 oncogene homolog, 3 inflammation related and one unknown gene product. Evaluating the common GO terms we find in all, except in the inflammation related gene products, terms related to apoptosis and more specific anti-apoptosis. The inflammation related gene products have terms linked to regulation of cell proliferation in common with BCL2_HUMAN.

Comparing the results by means of sequence similarity (Figure 4a, line), it becomes clear that apart from the orthologous gene products, all sequences have a similarity value below 0.5; for genes products not belonging to the Bcl-2 family the value is below 0.2. Therefore it is very difficult to define them as 'similar to BCL2_HUMAN' according to the sequence comparison; they were selected by ENGINE because of their similar description.

The second example, Q88869_MIDDV, is illustrated in Table 5, a collection of information about analogous gene products. Q88869_MIDDV also has a unique description and appears to be the best hit. This example was chosen because it is an example of a well described gene product from a non-model organism having non-electronic evidence for all its associated terms. Since Q88869_MIDDV is a short gene product (37 amino acids), there are no known protein domains reported (Table 5); a tblastn search found only fragments of other virus which have mostly GO associations to RNA polymerase activity and RNA processing (data not shown). However, with the help of ENGINE, and because the researcher had communicated some experimental information to GOA, we could find functionally analogous gene products with a similar GO term profile in organisms such as human, rat, mouse, *Arabidopsis*, fruitfly (as a representative of insects), and the protozoan cause of the sleeping disease, *Trypanosoma brucei; *a total of 10 different organisms. Q88869_MIDDV has general functional annotations such as 'transporter activity' and 'protein binding', is involved in more specific processes such as 'protein targeting' and 'endosome transport', and has the specific locus descriptions 'endosome' and 'trans-Golgi network transport vesicle membrane'. Functional analogues ranked 2 and 3 (a human and a rat endosomic gene product with a RasGTP effectors domain) have almost identical term profiles, as confirmed by their χ^2 ^– values. However, sequence similarity is very low (Figure 4b) and it would be improbable to find them using a sequence based approach. All other hits are involved in a kind of vesicle transport activity, which can be deduced from the kinds of domains those gene products contain, with some common directly associated terms and mostly common parental terms. This example demonstrates that ENGINE can assist a scientist with a gene product with experimental annotations to find functionally similar gene products, which could provide further information for future experiment planning.

**Table 5:** Example with Q88869_MIDDV

Rating	Symbol	UniProt	χ^2^	Common terms	Pfam	Organism	Local alignment	
							Hit length	Identity %
1	Q88869_MIDDV	Q88869	20080,7	54	No	Middelburg virus	37	100.0
2	O75901	O75901	19332,8	54	RA, RasGTP effectors	Homo sapiens	28	25.0
3	Pamci	O88869	17455,7	54	RA, RasGTP effectors	Rattus norvegicus	6	66.7
4	Tb09.244.2760	Q38CZ1	12733,8	44	-, Golgi dynamics	Trypanosoma brucei	36	27.8
5	Tb11.01.6880	Q381R2	12733,8	44	GP25L, Golgi dynamics	Trypanosoma brucei	44	22.7
6	TOM1_HUMAN	O60784	11529,8	35	VHS; membrane targeting	Homo sapiens	42	23.8
7	Vps28	Q9D1C8	11486,0	32	VPS28, Vacuolar protein sorting	Mus musculus	40	32.5
8	AT1G28490.1	Q946Y7	11360,8	32	SNARE, vesicular fusion	Arabidopsis thaliana	33	21.2
9	AT1G28490.2		11360,8	32	SNARE, vesicular fusion	Arabidopsisthaliana	21	23.8
10	AT5G16830	Q39233	11011,1	35	SNARE, vesicular fusion	Arabidopsis thaliana	27	29.6
11	AT5G46860.1	P93654	11011,1	35	SNARE, vesicular fusion	Arabidopsis thaliana	30	30.0
12	CG9308	Q9W2A6	11007,0	36	GP25L, Golgi dynamics	Drosophila melanogaster	14	28.6
13	AT5G26980.1	O65359	10926,6	34	SNARE, vesicular fusion	Arabidopsis thaliana	24	29.2
14	AT4G02195.1	Q9SWH4	10926,6	34	SNARE, vesicular fusion	Arabidopsis thaliana	24	33.3
15	AT5G26980.2		10926,6	34	SNARE, vesicular fusion	Arabidopsis thaliana	24	29.2
16	SPBC4F6.05c	O42707	10881,4	35	Lectin_leg-like	Schizosaccharomyces pombe	31	25.8
17	COPB_HUMAN	P53618	10873,7	39	No	Homo sapiens	23	30.4
18	VPS27	P40343	10803,4	33	VHS; membrane targeting	Saccharomyces cerevisiae	32	31.2
19	tlg1	Q9HGN3	10758,7	32	SNARE, vesicular fusion	Schizosaccharomyces pombe	38	18.4
20	ENT5	Q03769	10505,4	38	ENTH, membrane interacting	Saccharomyces cerevisiae	27	14.8
21	sft2	Q9P6K1	10398,2	33	SFT2, vesicular transport	Schizosaccharomyces pombe	11	36.4
22	Cope	O89079	10365,0	32	Coatomer_E, Golgi to ER transport	Mus musculus	29	27.6
23	Copb1	P23514	10363,2	37	Adaptin_N; coated vesicles	Rattus norvegicus	23	30.4
24	Tgoln2	Q4G0B6	10255,5	34	No	Rattus norvegicus	14	28.6
25	Vps26	P40336	10182,5	29	Vps26, Vacuolar protein sorting	Mus musculus	7	57.1
26	SPAC630.11	Q9UUH1	10182,0	30	Vps55, Vacuolar protein sorting	Schizosaccharomyces pombe	9	44.4
27	apl4	Q9UU81	10141,8	37	Adaptin_N; coated vesicles	Schizosaccharomyces pombe	17	35.3
28	apm1	Q9HFE5	10141,8	37	-, Clathrin coated vesicles	Schizosaccharomyces pombe	24	25
29	apl2	O43079	10141,8	37	Adaptin_N; coated vesicles	Schizosaccharomyces pombe	16	37.5
30	Q6DE70_XENLA	Q6DE70	10135,7	33	C2, Ca2+-dependent membrane-targeting	Xenopus laevis	8	50.0

The table contains the following information: The ranking of the potential functional analogues of Q88869_MIDDV according the χ^2 ^- value, the gene name and the UniProt accession number, the χ^2 ^- value, the number of common terms between a hit and Q88869_MIDDV, the Pfam name with short domain description the gene product belongs to, the organism the gene products is from, and the local alignment characteristics, such as hit length and the % identity of the hit, of the alignment between Q88869_MIDDV and the hit sequence.

In both test gene products we found hits in more than 10 different organisms, proving that ENGINE finds functionally similar gene products across several organisms, not only human or model organisms.

### Comparison with similar tools

ENGINE was compared with the GO-family tool, a member of the long list of tools using GO, which calculates a functional similarity measurement using the GO. The GO-family tool is part of the package called GOToolBox [24], that uses a statistical method to compare two gene products according to their descriptions via the GO and calculates a list of functionally similar gene products for a gene product of interest.

The main differences between the tools are:

- GO-family is limited to 7 organisms, all of them model organisms (*Arabidopsis thaliana, Caenorhabditis elegans, Drosophila melanogaster, Homo sapiens, Mus musculus, Rattus norvegicus, Saccharomyces cerevisiae*), whereas ENGINE compares the gene products from 13 model organisms with gene products of **all **available organisms within the GODB.

- GO-family uses the method of DICE distance (alias Sorensen, Czekanowski), giving double-weighting to the common terms, whereas ENGINE uses a χ^2 ^– approach, including a semantic measurement to weight the information content of each term used for the comparison.

A common search was performed with both tools for the BCL2_HUMAN gene product. Comparing only the 30 best hits shows that only 7 gene products are in common, and they all belong to the Pfam BCL2 protein family. Further, ENGINE finds 14 BCL2 family members, whereas GO-family finds 7; ENGINE's 30 best hits contain gene products from 8 different protein families, whereas the output from GO-family is distributed over 14 protein families; and ENGINE's output contains gene products from 8 different species, whereas the output of GO-family is from 4 different species.

## Discussion and conclusion

With ENGINE we demonstrate that GO has the potential to be used not only for analysis of biological data but also directly for comparative researches like the search for functional analogues we implemented in ENGINE. When compared with other similar tools, in particular with the GO-family tool, we find that ENGINE provides different and complementary information.

However, it is important to mention that although there is a large space of knowledge provided by the GO, there are still limitations for an optimal comparative analysis. Too many gene products and mainly from non-model organisms have low level and/or prevalently electronically evaluated associations. For this reasons too many gene products have an identical description: only additional experimental knowledge will differentiate those gene products from each other. There is also a certain redundancy for the same biological abstraction leading to a separation of the same biological function and therefore also to the separation of the corresponding gene products. This limitation of the GO has to be taken in account, also from the side of all other tools using this knowledge. Due to the fact that GO is daily updated, it is conceivable that most of these problems will progressively be reduced. It is worth to state again that additional effort is required to improve the GO by adding more experimental information for non-model organisms and really push the GO over the crucial limit for being used for optimal comparative analysis.

The run of ENGINE over the GRID showed to be highly effective: the functional analogy search was divided in a large number of jobs and distributed over the INFN production GRID to reduce the processing time from about 5 years on a single machine to about 3 days occupying up to 950 worker nodes. The acceleration of the process was measured to be 580 times. The prototype framework used by ENGINE for the remote installation of the MySQL DB and of the Perl Library, the application monitor performed by JAM and the job submitting mechanism showed to be highly reliable and quite general to be applied in other bioinformatics applications as well as in other research fields.

Recently we have been working on a new version of ENGINE applicable to the steadily increasing whole set of more than 2 millions gene products in the GODB using the GRID infrastructure of EGEE, and, importantly, an efficient updating procedure to profit from the new monthly GODB versions and increasing knowledge to be able to provide with the same frequency the new search data.

## Authors' contributions

AT was responsible for the feasibility study, the development of the Perl script to calculate the statistical comparison of the gene products and writing part of the manuscript. GD was responsible for the development of the GRID technology part and writing part of the manuscript. FL was responsible for adjustment and update of the gene ontology database. GM was involved in the development of the GRID technology part and writing the manuscript. AG guided the project, was involved in the development of the Perl script to calculate the statistical comparison of the gene products and writing the manuscript. All authors read and approved the final manuscript.
